# Supplementary material for: Printed Soft Sensor with Passivation Layers for the Detection of Object Slippage by a Robotic Gripper
Source: Micromachines (Basel). 2020 Oct 8;11(10):927. doi: 10.3390/mi11100927 (PMC7599578; doi:10.3390/mi11100927)
Supplement: Supplementary file 1 [file micromachines-11-00927-s001.pdf]

micromachines

Supplementary materials

Printed Soft Sensor with Passivation Layers for the Detection of Object Slippage by a Robotic Gripper

Reo Miura [1], Tomohito Sekine [1,*], Yi-Fei Wang [1], Jinseo Hong [1], Yushi Watanabe [1], Keita Ito [1], Yoshinori Shouji [1], Yasunori Takeda [1], Daisuke Kumaki [1], Fabrice Domingues Dos Santos [2], Atsushi Miyabo [3] and Shizuo Tokito [1,*]

[1] Research Center for Organic Electronics (ROEL), Graduate School of Science and Engineering, Yamagata University, 3-4-16, Jonan, Yonezawa, Yamagata 992-8510, Japan; tnh99114@st.yamagata-u.ac.jp (R.M.); wang@yz.yamagata-u.ac.jp (Y.-F.W.); jinseo.hong@yz.yamagata-u.ac.jp (J.H.); tnn26268@st.yamagata-u.ac.jp (Y.W.); tae33543@st.yamagata-u.ac.jp (K.I.); txc00320@st.yamagata-u.ac.jp (Y.S.); y.takeda@yz.yamagata-u.ac.jp (Y.T.); d_kumaki@yz.yamagata-u.ac.jp (D.K.)

[2] Piezotech S. A. S., Arkema-CRRA, Rue Henri Moissan, 63493 Pierre-Benite Cedex, France; fabrice.domingues-dos-santos@arkema.com

[3] Arkema K. K., 2-2-2 Uchisaiwaicho, Chiyoda-ku, Tokyo 100-0011, Japan; atsushi.miyabo@arkema.com

* Correspondence: tomohito@yz.yamagata-u.ac.jp (T.S.); tokito@yz.yamagata-u.ac.jp (S.T.)

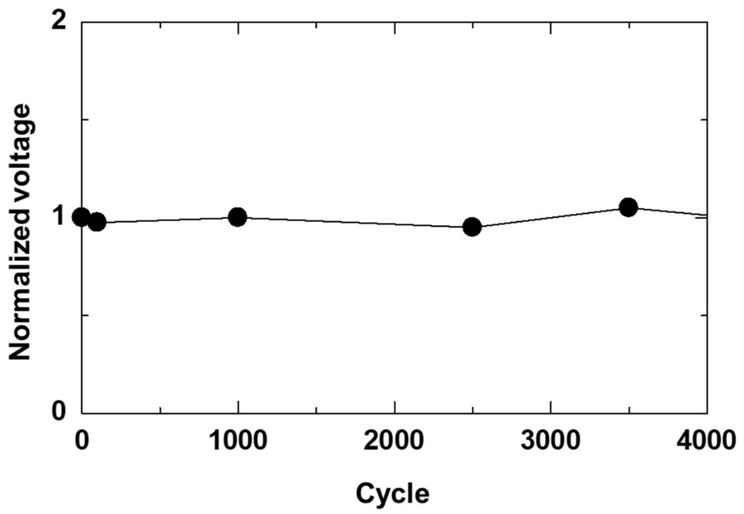

Figure S1. A mechanical fatigue of our sensor as a normalized voltage during long-term cycling for 3500 cycles.

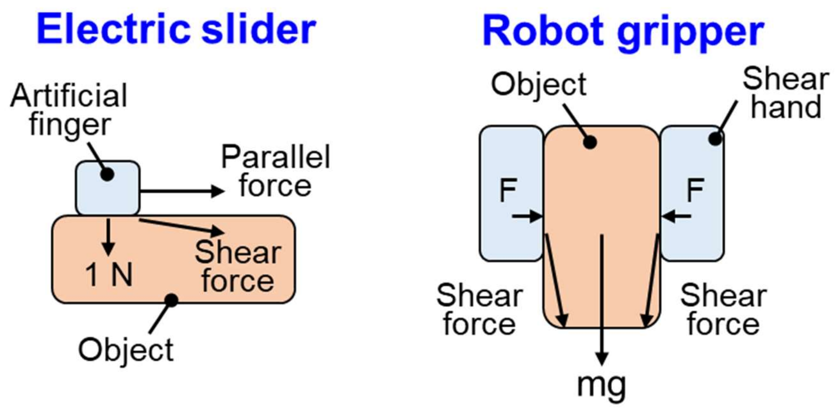

Figure S2. Shear force occurring of shear force in testing of Figure 4a and d.

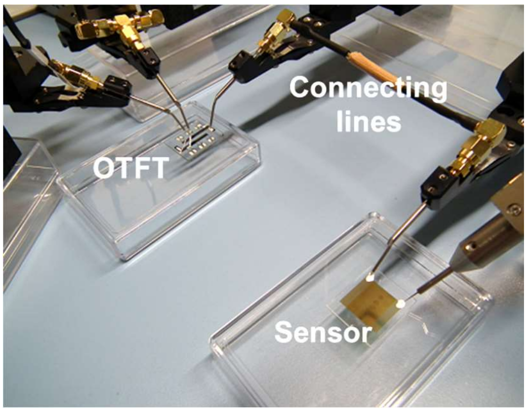

Figure S3. A connection system with the sensor and OTFT as the amplifier circuit.
